# Switchable crossed spin conductance in a graphene-based junction: The role of spin-orbit coupling

**DOI:** 10.1038/s41598-020-58799-6

**Published:** 2020-02-06

**Authors:** Razieh Beiranvand, Hossein Hamzehpour

**Affiliations:** 1 Physics Group, Department of Basic Science, Ayatollah Boroujerdi University, Boroujerd, Iran; 2 Department of Physics, K.N. Toosi University of Technology, Tehran, 15875-4416 Iran

**Keywords:** Applied physics, Condensed-matter physics

## Abstract

We theoretically investigate the crossed spin conductance (CSC) of a graphene-based heterostructure consists of ferromagnet, Rashba spin-orbit and superconductor regions. Using Dirac Bogoliubov-de Gennes formalism in the ballistic regime, we show that in the presence of Rashba spin-orbit coupling there are an anomalous crossed Andreev reection and spin-ipped co-tunneling in the process of quantum transport. We demonstrate that the CSC can be reversed with respect to charge conductance by tuning the Rashba spin-orbit coupling which experimentally can be adjusted by the applied perpendicular electric field on the graphene sheet. This feature in addition to a long spin relaxation time of Dirac fermions in graphene proposes designing a device with a non-local spin switch which is crucial for spintronics circuits.

## Introduction

Hybrid structures of superconductivity have a suitable potential to use in the future technology^1^. A wide variety of interesting phenomena such as topological superconductivity^2^ Majorana fermions^3–5^, topological quantum computation^6^ require superconductivity. Also, a major part of growing field of spintronics needs superconductivity^7^. More than 15 years, scientist have been tried to find a way for combining superconductivity and spintronics to make a long-range spin-polarized super-currents. Long spin relaxation time of Dirac fermions in graphene made it an important candidate for using in such a device^8,9^. Graphene not only has a simple structure, but also can be easily prepared in experiments with different properties. Superconductivity (S) and ferromagnetism (F) can be induced into graphene by means of proximity effect^10–14^. Designing hybrid structures involving S and F regions make graphene a very good host to explore the fascinating phenomena such as Klein tunneling^15,16^, supercurrent *π*-junction^17^ and spin-triplet correlation^18^. In contrast ot ordinary superconductors, the superconducting junction of graphene has the capability of reflecting an incident electron as a hole with a specular path. This process is known as Andreev reflection and the missing charge of 2*e* enters the superconductor as a Cooper pair^19,20^. The low-energy excitations of graphene near the K and K′ points of the Brillouin zone are governed by a 2D massless Dirac Hamiltonian^21,22^. The linear conduction and valence bands cross the Fermi level at these points. Because of these unique properties, graphene attracts huge attentions of scientists from fundamental to the technical point of view^23^. The Fermi level in graphene in contrast to ordinary conductors or semiconductors, can be also tuned by a gate voltage^15,24^.

Spin-Orbit interaction has intrinsic or extrinsic origin in the graphene^25–28^. The first one which is known as Dresselhaus spin-orbit (DSO) interaction^29^ comes from the spin dependent second neighbor hopping. The last one which is known as Rashba spin-orbit (RSO) interaction^30^, arises from a perpendicular electric field or interactions with substrate^31^. The RSO interaction which is induced by proximity to a tungsten disulphide substrate^28^ confirmed experimentally to be ~17 meV. The DSO interaction which is responsible to create a small gap in the band structure, predicted to be quite small in comparison with the larger effects like RSO interaction^32^. So, in this paper we ignore the DSO interaction and just focus on the RSO interaction.

When two metallic leads are connected to a superconductor, non local Andreev reflection processes can be generated. The incident quasi-particle at the interface of one lead can be reflected as a quasi-hole in the other lead, giving rise to a negative non local conductance. This so-called crossed Andreev reflection (CAR) competes with elastic co-tunneling (CT). The graphene-based junctions contain RSO interaction in their interfaces predicted to show odd-frequency triplet correlations^33^, maximum spin Seebeck effect^34^, and tunable magneto-resistance^35^. In our previous work^36^, we show that the RSO interaction in the interfaces of F-S-F graphene junction generates the anomalous CAR and spin-flipped CT. These effects are the direct signature of breaking translational symmetry at the interfaces due to the RSO interaction and penetrating equal-spin triplet correlation into ferromagnetic region. Using gate voltage and tuning Fermi level, the sign of charge conductance in the ferromagnetic drain lead (F_2_) can be negative (See more details in refs. ^33,36^.

## Methods and Discussions

In this letter, we proposed an experimental accessible device to generate negative crossed spin conductance (CSC) and tunable crossed charge conductance (CCC). We extend the Landauer^37^ and Blonder-Tinkham-Klapwijk^38^ formalism to derive two formulas for the CCC and CSC in the drain lead. We also show that the sign of the spin conductance can be switch from positive to negative by changing RSO interaction which is experimentally possible by tuning perpendicular electric field. This property in addition to the long spin relaxation time of Dirac fermions makes this set up very suitable for spintronics circuits. It is known that the typically *p*-wave superconductors are rare in nature. Normal impurities can easily break the Cooper pairs in *p*–wave and *d*–wave superconductors. But, the s-wave superconductors are robust against impurities and much more common in nature. So we consider an s-wave superconductor in the junction.

**Figure 1:**
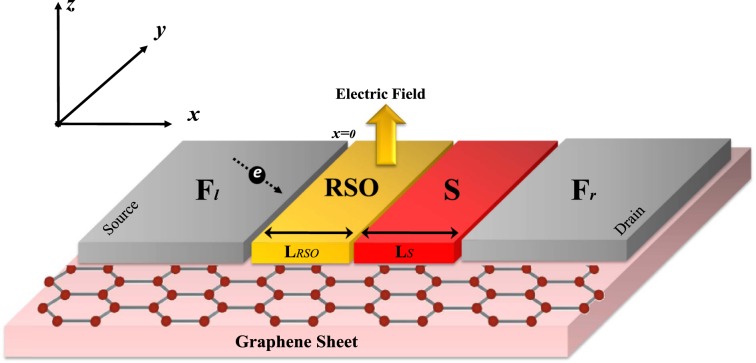
Schematic of the graphene based F-RSO-S-F heterostructure. The sheet of graphene resides in the *x* − *y* plane. The length of the RSO and S regions are denoted by L_RSO_ and L_S_. The exchange field of the ferromagnetic source and drain leads (${\overrightarrow{h}}_{l,r}$) are assumed fixed along the *z* axis. This graph drawn by LibreOffice 6.3.4, https://www.libreoffice.org/download/download/?type=deb-x86_64amp;version=6.3.4amp;lang=en-US.

We consider a sheet of graphene in the *x* − *y* plane. We suppose that ferromagnetism, singlet superconductivity and RSO interaction are applied on the graphene by proximity. As shown in Fig. 1, the left ferromagnetic lead acts as a source of charge and spin carriers and the right one acts as a drain to collect quasi-particles. We assume the interface of each region is rigid and disorder free which correspond to the ballistic limit^5,15,17–19,33,34,36^ because in a typical experimental situation, ballistic propagations can be spoiled by edge disorders.

The low-energy of quasi-particles of such a device describes by Dirac Bogoliubov-de Gennes DBdG equation

1 $$(\begin{array}{cc}{H}_{D}+{H}_{i}-{\mu }^{i} & \Delta {e}^{i\phi }\\ {\Delta }^{\ast }{e}^{-i\phi } & {\mu }^{i}-{\mathcal{T}}\,[{H}_{D}-{H}_{i}]{{\mathcal{T}}}^{-1}\end{array})(\begin{array}{c}u\\ v\end{array})=\varepsilon (\begin{array}{c}u\\ v\end{array}),$$

The original DBdG Hamiltonian is a 16 × 16 matrix in the presence of an arbitrary direction of magnetization and spin-orbit interaction. However, graphene has another degrees of freedom, valley degeneracy which is responsible to electron-hole conversion. Due to the valley degeneracy in graphene, the Hamiltonian reduces to a 8 × 8 matrix. Nonetheless, one can use different basis sets that consequently change the form of Hamiltonian without affecting final physical results and conclusions. In our representation, the hole part is time-reversed of the electron part which denoted it by ${\mathscr{T}}$ ^19^, and $\varepsilon$ is the energy of quasi-particles with respect to the Fermi energy of each region, *μ*^*i*^. Also, ${H}_{D}=\hslash {v}_{F}{s}_{0}\otimes ({\sigma }_{x}{k}_{x}+i{\sigma }_{y}{k}_{y})$ is the two-dimensional Dirac Hamiltonian which governs on the carriers of graphene with ${v}_{F}$ being the Fermi velocity. ${s}_{x,y,z}$ and ${\sigma }_{x,y,z}$ are Pauli matrices, acting on spin and pseudo-spin degrees of freedom, respectively. The amplitude and phase of singlet Cooper pairs described by $\Delta ={\Delta }_{0}\Theta (x-{L}_{RSO})\Theta ({L}_{s}+{L}_{RSO}-x){s}_{0}\otimes {\sigma }_{0}$ and $\phi$, respectively. Here, Δ_0_ is the superconducting gap in zero temperature and $\Theta (x)$ denotes the step function. As far as ${\lambda }_{F}^{S}\ll \{{\lambda }_{F}^{RSO},{\lambda }_{F}^{F}\}$, this step function assumption is valid^39^ in which ${\lambda }_{F}^{F}$, ${\lambda }_{F}^{RSO}$ and ${\lambda }_{F}^{S}$ are the value of Fermi wave lengths in ferromagnetic leads, Rashba spin-orbit region and superconductor, respectively. Although the smooth change at the junction which is occurred in realistic systems, can alter the results qualitatively but not the conclusions. Also, $u(v)$ is the electron (hole) part of DBdG wave function in the electron-hole space. The corresponding Hamiltonians in each region may written as,

2 $${H}_{i}=(\begin{array}{ll}{H}_{F}={h}_{l}({s}_{z}\otimes {\sigma }_{0}) & x\le 0\\ {H}_{RSO}=\lambda ({s}_{y}\otimes {\sigma }_{x}-{s}_{x}\otimes {\sigma }_{y}) & 0\le x\le {L}_{RSO}\\ {H}_{S}=-\,{U}_{0}({s}_{0}\otimes {\sigma }_{0}) & {L}_{RSO}\le x\le {L}_{S}+{L}_{RSO}\\ {H}_{F}={h}_{r}({s}_{z}\otimes {\sigma }_{0}) & {L}_{S}+{L}_{RSO}\le x\end{array}.$$

The magnetic exchange field in source and drain leads (F_*l*_ and F_*r*_) represents by *h*_*l*_ and *h*_*r*_, respectively. Their directions fixed along the *z* axis without loss of generality. This choice makes the spin-dependent analyses of scattering process clear. *λ* is the strength of RSO interaction^32^. *U*_0_ is an electrostatic potential which is necessary to prepare enough density of states for induced superconductivity. So we assume heavily doped approximation, ${U}_{0}\gg \{{\Delta }_{0},\varepsilon \}$, which is experimentally suitable^19,39^. The length of RSO and S regions are *L*_*RSO*_ and *L*_*S*_, respectively. The eigenvalues of each region are obtained by diagonalizing Eq. (1) as,

3 $$\varepsilon =(\begin{array}{ll}\pm {\mu }^{{F}_{l}}\pm \sqrt{{({k}_{x}^{{F}_{l}})}^{2}+{q}_{n}^{2}}\pm {h}_{l} & x\le 0\\ \pm {\mu }^{RSO}\pm \sqrt{{({k}_{x}^{RSO})}^{2}+{q}_{n}^{2}+{\lambda }^{2}}\pm \lambda  & 0\le x\le {L}_{RSO}\\ \pm \sqrt{{({\mu }^{S}+{U}_{0}\pm \sqrt{{({k}_{x}^{S})}^{2}+{q}_{n}^{2}})}^{2}+|{\Delta }_{0}{|}^{2}} & {L}_{RSO}\le x\le {L}_{RSO}+{L}_{S}\\ \pm {\mu }_{r}^{F}\pm \sqrt{{({k}_{x}^{{F}_{r}})}^{2}+{q}_{n}^{2}}\pm {h}_{r} & {L}_{RSO}+{L}_{S}\le x\end{array}.$$

The associated wave functions are given in Appendix and refs. ^33,34,36^. The energy and transverse component of wave vector, *q*_*n*_, in each region is conserved during the scattering process whereas the longitudinal component, ${k}_{x}^{i}$ acquires different value according to Eq. (3).

The scattering processes due to the differential voltage bias considered as follow: A spin-up electron with wave function ${\psi }_{e,\uparrow }^{{F}_{l},+}$ hits the RSO interface from the source lead at $x=0$. This particle can be reflected as a spin-down hole, ${\psi }_{h,\downarrow }^{{F}_{l},-}$, (spin-up, ${\psi }_{h,\uparrow }^{{F}_{l},-}$,) into the source lead due to conventional (anomalous) Andreev process. Also, it can be reflected as an electron due to normal (spin conserved) or spin-flipped reflection. So, the total wave function in the source lead is:

4 $$\begin{array}{rcl}{\Psi }^{{F}_{l}}(x) & = & {\psi }_{e,\uparrow }^{{F}_{l},+}(x)+{r}_{N}^{\uparrow }{\psi }_{e,\uparrow }^{{F}_{l},-}(x)+{r}_{N}^{\downarrow }{\psi }_{e,\downarrow }^{{F}_{l},-}(x)\\  &  & +\,{r}_{A}^{\downarrow }{\psi }_{h,\downarrow }^{{F}_{l},-}(x)+{r}_{A}^{\uparrow }{\psi }_{h,\uparrow }^{{F}_{l},-}(x).\end{array}$$

Moreover, it can be transfered into the ferromagnetic drain lead as an electron which is called co-tunneling (CT) process or as a hole which is called crossed Andreev reflection (CAR). So, in the scattering process into drain lead we have four probabilities: spin-preserved CT $|{t}_{e}^{\uparrow }{|}^{2}$, spin-flipped co-tunneling $|{t}_{e}^{\downarrow }{|}^{2}$, conventional CAR $|{t}_{h}^{\downarrow }{|}^{2}$ and anomalous CAR $|{t}_{h}^{\uparrow }{|}^{2}$. The total wave function in the drain lead which is our interest reads,

5 $${\Psi }^{{F}_{r}}(x)={t}_{e}^{\uparrow }{\psi }_{e,\uparrow }^{{F}_{r},+}(x)+{t}_{e}^{\downarrow }{\psi }_{e,\downarrow }^{{F}_{r},+}(x)+{t}_{h}^{\downarrow }{\psi }_{h,\downarrow }^{{F}_{r},+}(x)+{t}_{h}^{\uparrow }{\psi }_{h,\uparrow }^{{F}_{r},+}(x).$$

Furthermore, the total wave function in RSO (${\Psi }^{RSO}(x)$) and S (${\Psi }^{S}(x)$) regions can be calculated in a similar way^33^. In the RSO region, the wave function is split into electron and hole sector, and these two are not coupled. When an incoming electron is entering the RSO region and reaching the RSO/S interface, it is converted into a hole that travels back into the ferromagnetic contact. All of the possibilities and scattering processes are explained in previous works in details^33^. All wave functions should satisfy the boundary conditions as follow, to calculate the probability amplitudes.

6 $$(\begin{array}{ll}{\Psi }^{{F}_{l}}(x)={\Psi }^{RSO}(x) & x=0\\ {\Psi }^{RSO}(x)={\Psi }^{S}(x) & x={L}_{RSO}\\ {\Psi }^{S}(x)={\Psi }^{{F}_{r}}(x) & x={L}_{RSO}+{L}_{S}\end{array}.$$

**Figure 2:**
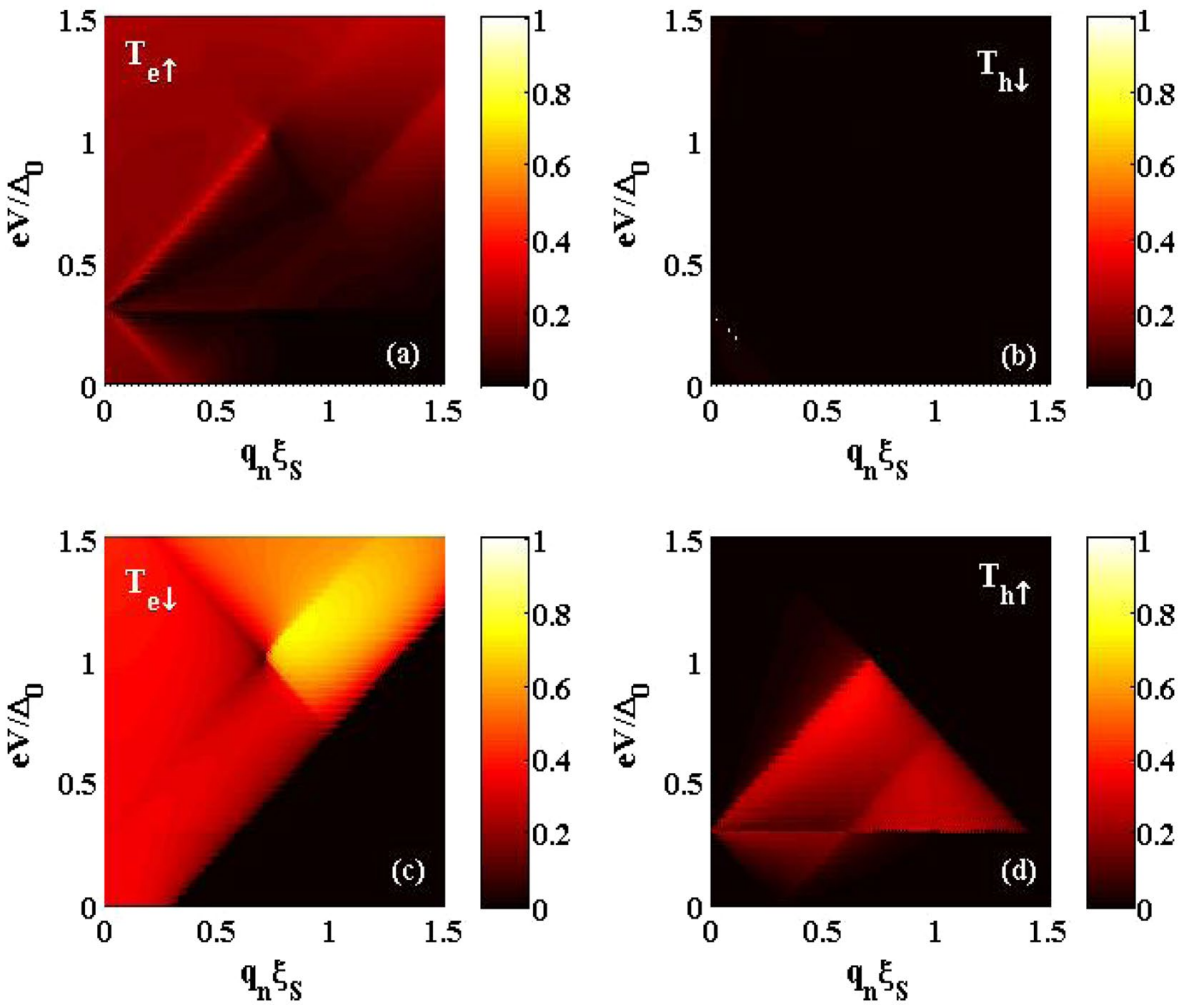
(**a**) Spin-preserved cotunneling probability $|{t}_{e}^{\uparrow }{|}^{2}$, (**b**) conventional crossed Andreev reflection probability $|{t}_{h}^{\downarrow }{|}^{2}$, (**c**) spin-flipped cotunneling probability $|{t}_{e}^{\downarrow }{|}^{2}$, (**d**) anomalous Crossed Andreev reflection probability $|{t}_{h}^{\downarrow }{|}^{2}$. The probabilities are plotted vs the transverse component of wave vector ${q}_{n}$ and voltage bias across the junction *eV*. We set ${\mu }^{{{\rm{F}}}_{l}}={\mu }^{{{\rm{F}}}_{r}}={\Delta }_{0},{h}_{l}={h}_{r}=0.7{\Delta }_{0},{\mu }^{RSO}=8{\Delta }_{0},\lambda =1.5{\Delta }_{0},{L}_{{\rm{RSO}}}=0.4{\xi }_{S},{L}_{{\rm{S}}}=0.8{\xi }_{S}$. The plots drawn by Python 3.8.1, https://www.python.org/ Python 3.8.1.

We normalize the lengths by superconducting coherence length ${\xi }_{S}=\hslash {v}_{F}/{\Delta }_{0}$ and energies by superconducting gap at zero temperature ${\Delta }_{0}$. In the absence of RSO interaction anomalous Andreev and spin-flipped reflections in the source lead and anomalous CAR and spin-flipped CT in the drain lead disappear. This means by turning on the RSO interaction non-locally in RSO region, these novel process which are very important in transport phenomena such as charge and spin conductance will be present. The probabilities related to drain lead, *F*_*r*_, are shown in Fig. 2. To show the effect of RSO interaction very clear, we set ${L}_{RSO}=0.4{\xi }_{S}$ and ${L}_{S}=0.8{\xi }_{S}$, which is smaller than the coherence length of Cooper pair in the superconductor. The other related parameters are set as: ${\mu }_{{F}_{l}}={\mu }_{{F}_{r}}={\Delta }_{0},{\mu }_{RSO}=8{\Delta }_{0},{h}_{l}={h}_{r}=0.7{\Delta }_{0}$ and $\lambda =1.5{\Delta }_{0}$. As shown in Fig. 2, the anomalous CAR which is very important in our setup, isolated in the space of energy-transverse momentum whereas the conventional CAR is blocked. This make its detection easier. If one uses Nb electrode as a superconductor, the energy gap is ~1 *meV* and coherence length is ~10 *nm*^40^. These values lead to *L*_*S*_ = 8 *nm*, ${L}_{RSO}=0.4\,nm$, $\lambda =1.5\,meV$, ${h}_{l}={h}_{r}=0.7\,meV$, ${\mu }_{RSO}=8\,meV$ and ${\mu }_{{F}_{l}}={\mu }_{{F}_{r}}=1\,meV$. So, in a realistic experiment we predict the anomalous CAR can be detected in $\varepsilon \le {\Delta }_{0}$.

We extend the Landauer^37^ and Blonder-Tinkham-Klapwijk^38^ formalism to derive two formulas for the CSC and CCC in the drain lead. The extended version of BTK formula for the charge transport is,

7 $$G=\int d{q}_{n}\,\sum _{s=\uparrow ,\downarrow }{G}_{s}({|{t}_{e}^{s}|}^{2}-{|{t}_{h}^{s}|}^{2}),$$

where ${G}_{\uparrow ,\downarrow }=2{e}^{2}W|\varepsilon +{\mu }_{l}\pm {h}_{l}|/\pi \hslash$ is the ballistic conductance of the ferromagnetic source lead. The summation over s index indicates that the scattering process of electron with spin-down must be take into account as well. Here, W is the width of the junction. Furthermore, anomalous CAR and spin-flipped CT can alter the formula of spin conductance^41,42^ since they carry spin as well. The anomalous CAR which carry a spin-up electron in our scenario decreases the spin conductance whereas th spin-flipped CT increases it. So, the CSC of the junction can be read as

8 $${G}_{S}=\int d{q}_{n}\sum _{s=\uparrow ,\downarrow }{G}_{s}((|{t}_{e}^{\uparrow }{|}^{2}-|{t}_{e}^{\downarrow }{|}^{2})-({|{t}_{h}^{\uparrow }|}^{2}-{|{t}_{h}^{\downarrow }|}^{2})),$$

**Figure 3:**
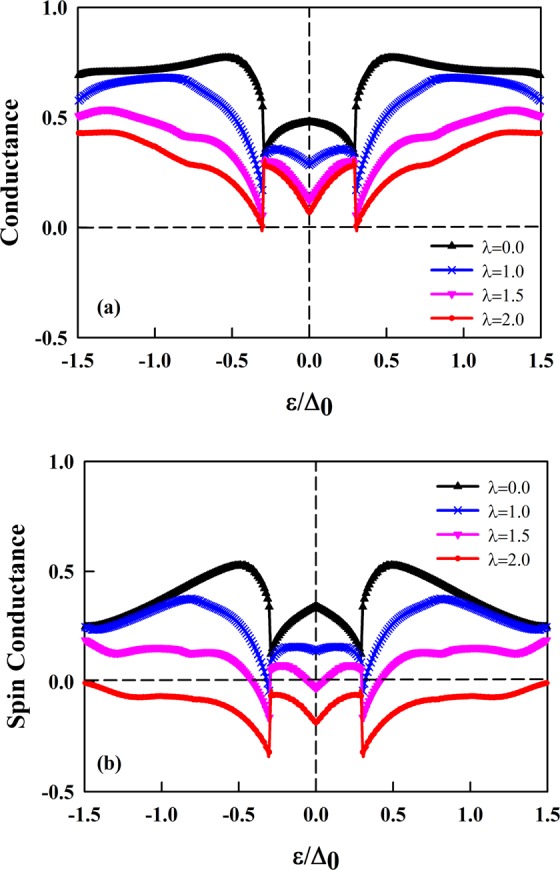
Crossed charge conductance (**a**) and crossed spin conductance (**b**) associated with the probabilities presented in Fig. 2. The conductances are normalized by ${G}_{0}={G}_{\uparrow }+{G}_{\downarrow }$. The RSO interaction varies between 0 to 2.0 meV.

An important consequence of the presence of RSO interaction is the appearance of negative CSC that is completely non-local. In Fig. 3, the CCC and CSC are calculated using Eqs. (7, 8) with the input values same as Fig. 2.

The application of RSO interaction can completely suppress the values of junction’s conductance, leading to negative CSC and positive CCC simultaneously. When the current approaches the right ferromagnetic lead in the junction, it passes through with no reflection. This is a manifestation of the CCC. The passed carriers have a down-spin or up-spin direction. Negative CSC means that all carriers have similar spin directions. As the *λ* appears, the anomalous CAR and spin-flipped CT grow to became dominant in transport phenomena. This made the CSC to be negative while the CCC keeps its positive values. In such a case, the junction’s conductance changes in discrete steps when the gate voltage is varied. It is found that, when the gate voltage reaches to the charge neutrality point, both CCC and CSC are minimum. As shown in Fig. 3, there are two points in which the value of CSC is zero. This type of discontinuity and its related physics was described in previous works in detail^19,33^. During the Andreev processes in metallic junction, the reflected hole was created in the conduction band in a retro-reflection type. In graphene-based junction, the reflected hole can be created either in conduction band or valence band according to its energy with respect to Fermi level (The reflected hole in the valence band is a specular type). At the point where these two types convert to each other, the mentioned discontinuity would happen.

**Figure 4:**
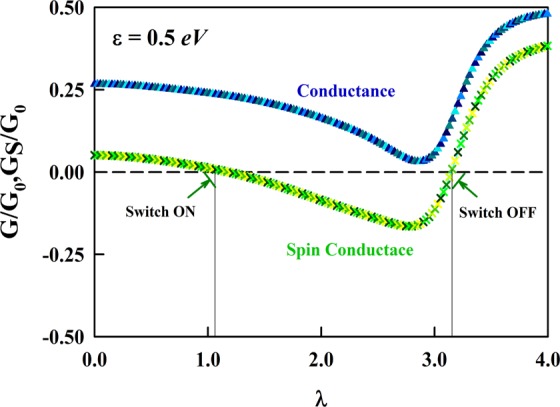
Crossed charge and spin conductances versus *λ*. Tuning RSO interaction leads to negative crossed spin conductances whereas the charge conductance keep its value positive. The input values are same as Fig. 2 and gate voltage set to be 0.5 eV.

Interestingly, changing the value of RSO term alters the sign of CSC from positive to negative values and vice versa, as shown in Fig. 4. The distance between the On and OFF switch is proportional to the RSO term and other tunable parameters in graphene junction. Because the spin relaxation time in graphene is long enough, the quantum states can be tuned with the strength of the RSO interactions. This property also allows the anomalous CAR and negative CSC to be probed experimentally in graphene junction.

## Conclusions

Motivated by recent achievements in the induction of RSO interaction into a graphene sheet, we have theoretically studied quantum transport properties of a junction consists of ferromagnets, singlet superconductors and RSO interaction. As known, it is possible to change the RSO term non-locally in graphene by applying electric field. This situation provides a spin-switch device which has a capability to block the current with the specific spin direction. Our results demonstrate that in the presence of RSO interaction, there are new types of reflection and transmission probabilities. Among them, those who live in drain lead are our interest because of their non-local nature. Spin-flipped CT and anomalous CAR have negative roles in CSC. Due to these interesting capabilities which can be very crucial in spintronics, graphene junctions may, in principle, be used for spin-current control or spin orbit sensing applications in superconducting spintronics.

## Appendix

The wavefunctions associated with the dispersion relation in the F region are:

9 $$\begin{array}{rcl}{\psi }_{e,\uparrow }^{{\rm{F}},\pm }(x) & = & {({{\bf{0}}}^{{\bf{2}}},1,\pm {e}^{\pm i{\alpha }_{\uparrow }^{e}},{{\bf{0}}}^{{\bf{4}}})}^{{\bf{T}}}{e}^{\pm i{k}_{x,\uparrow }^{{\rm{F}},e}x},\\ {\psi }_{e,\downarrow }^{{\rm{F}},\pm }(x) & = & {(1,\pm {e}^{\pm i{\alpha }_{\downarrow }^{e}},{{\bf{0}}}^{{\bf{2}}},{{\bf{0}}}^{{\bf{4}}})}^{{\bf{T}}}{e}^{\pm i{k}_{x,\downarrow }^{{\rm{F}},e}x},\\ {\psi }_{h,\uparrow }^{{\rm{F}},\pm }(x) & = & {({{\bf{0}}}^{{\bf{4}}},1,\mp {e}^{\pm i{\alpha }_{\uparrow }^{h}},{{\bf{0}}}^{{\bf{2}}})}^{{\bf{T}}}{e}^{\pm i{k}_{x,\uparrow }^{{\rm{F}},h}x},\\ {\psi }_{h,\downarrow }^{{\rm{F}},\pm }(x) & = & {({{\bf{0}}}^{{\bf{4}}},{{\bf{0}}}^{{\bf{2}}},1,\mp {e}^{i\pm {\alpha }_{\downarrow }^{h}})}^{{\bf{T}}}{e}^{\pm i{k}_{x,\downarrow }^{{\rm{F}},h}x},\end{array}$$

where **0**^**n**^ represents a 1 × *n* matrix with only zero entries and **T** is a transpose operator.

The wavefunctions associated with the eigenvalues given in the text can be expressed by:

10 $$\begin{array}{rcl}{\psi }_{e,\eta =+1}^{{\rm{RSO}},\pm }(x) & = & {(\mp i{f}_{+}^{e}{e}^{\mp i{\theta }_{+}^{e}},-i,1,\pm {f}_{+}^{e}{e}^{\pm i{\theta }_{+}^{e}},{{\bf{0}}}^{{\bf{4}}})}^{{\bf{T}}}{e}^{\pm i{k}_{x,+}^{{\rm{RSO}},e}x}\\ {\psi }_{e,\eta =-1}^{{\rm{RSO}},\pm }(x) & = & {(\pm {f}_{-}^{e}{e}^{\mp i{\theta }_{-}^{e}},1,-i,\mp i{f}_{-}^{e}{e}^{\pm i{\theta }_{-}^{e}},{{\bf{0}}}^{{\bf{4}}})}^{{\bf{T}}}{e}^{\pm i{k}_{x,-}^{{\rm{RSO}},e}x}\\ {\psi }_{h,\eta =+1}^{{\rm{RSO}},\pm }(x) & = & {({{\bf{0}}}^{{\bf{4}}},\mp i{f}_{+}^{h}{e}^{\mp i{\theta }_{+}^{h}},-i,1,\pm {f}_{+}^{h}{e}^{\pm i{\theta }_{+}^{h}})}^{{\bf{T}}}{e}^{\pm i{k}_{x,+}^{{\rm{RSO}},h}x}\\ {\psi }_{h,\eta =-1}^{{\rm{RSO}},\pm }(x) & = & {({{\bf{0}}}^{{\bf{4}}},\pm {f}_{-}^{h}{e}^{\mp i{\theta }_{-}^{h}},1,-i,\mp i{f}_{-}^{h}{e}^{\pm i{\theta }_{-}^{h}})}^{{\bf{T}}}{e}^{\pm i{k}_{x,-}^{{\rm{RSO}},h}x}\end{array}.$$

here the definition of auxiliary parameters are:

11 $$\left\{\begin{array}{rcl}{f}_{\eta }^{e} & = & \sqrt{1+2\eta \lambda {({\mu }^{{\rm{RSO}}}+\varepsilon )}^{-1}}\\ {f}_{\eta }^{h} & = & \sqrt{1+2\eta \lambda {({\mu }^{{\rm{RSO}}}-\varepsilon )}^{-1}}\end{array}\right.,$$

and

12 $$\left\{\begin{array}{rcl}{\theta }_{\eta }^{e} & = & \arctan (\frac{{q}_{n}}{{k}_{x,\eta }^{{\rm{RSO}},e}})\\ {\theta }_{\eta }^{h} & = & \arctan (\frac{{q}_{n}}{{k}_{x,\eta }^{{\rm{RSO}},h}})\end{array}\right.,$$

where ${\theta }_{\eta }^{e(h)}$ are the electron and hole propagation angles in the region with spin orbit interaction.

The wavefunctions in the superconducting region are given by:

13 $$\begin{array}{rcl}{\psi }_{e,\kappa =1}^{{\rm{S}},\pm }(x) & = & {({e}^{+i\beta },\pm {e}^{+i\beta },{{\bf{0}}}^{{\bf{2}}},{e}^{-i\phi },\pm {e}^{-i\phi },{{\bf{0}}}^{{\bf{2}}})}^{{\bf{T}}}{e}^{\pm i{k}_{x}^{{\rm{S}},e}x}\\ {\psi }_{e,\kappa =2}^{{\rm{S}},\pm }(x) & = & {({{\bf{0}}}^{{\bf{2}}},{e}^{+i\beta },\pm {e}^{+i\beta },{{\bf{0}}}^{{\bf{2}}},{e}^{-i\phi },\pm {e}^{-i\phi })}^{{\bf{T}}}{e}^{\pm i{k}_{x}^{{\rm{S}},e}x}\\ {\psi }_{h,\kappa =1}^{{\rm{S}},\pm }(x) & = & {({e}^{-i\beta },\mp {e}^{-i\beta },{{\bf{0}}}^{{\bf{2}}},{e}^{-i\phi },\mp {e}^{-i\phi },{{\bf{0}}}^{{\bf{2}}})}^{{\bf{T}}}{e}^{\pm i{k}_{x}^{{\rm{S}},h}x}\\ {\psi }_{h,\kappa =2}^{{\rm{S}},\pm }(x) & = & {({{\bf{0}}}^{{\bf{2}}},{e}^{-i\beta },\mp {e}^{-i\beta },{{\bf{0}}}^{{\bf{2}}},{e}^{-i\phi },\mp {e}^{-i\phi })}^{{\bf{T}}}{e}^{\pm i{k}_{x}^{{\rm{S}},h}x}\end{array}.$$

The parameter *β* is responsible for the electron-hole conversions at the interface RSO-S and depends on the superconducting gap:

14 $$\beta =\left\{\begin{array}{ll}+\,\arccos (\varepsilon /{\Delta }_{0}) & \varepsilon \le {\Delta }_{0}\\ -i\,{\rm{arccosh}}(\varepsilon /{\Delta }_{0}) & \varepsilon \ge {\Delta }_{0}\end{array}\right..$$
